# Mercury and selenium in three fish species from a dam 20 months after a mine-tailing spill in the SE Gulf of California ecoregion, Mexico

**DOI:** 10.1007/s11356-023-31487-4

**Published:** 2023-12-20

**Authors:** Federico Páez-Osuna, Magdalena E. Bergés-Tiznado, Gladys Valencia-Castañeda, Marcela G. Fregoso-López, Jesús A. León-Cañedo, Juan F. Fierro-Sañudo, Javier Ramírez-Rochín

**Affiliations:** 1 Universidad Nacional Autónoma de México, Instituto de Ciencias del Mar y Limnología, Unidad Académica Mazatlán, P.O. Box 811, 82000 Mazatlán, Sinaloa Mexico; 2 Miembro de El Colegio de Sinaloa, Antonio Rosales 435 Poniente, Culiacán, Sinaloa Mexico; 3 Unidad Académica de Ingeniería en Tecnología Ambiental, Universidad Politécnica de Sinaloa, Carretera Municipal Libre Mazatlán-Higueras Km. 3, C.P. 82199 Mazatlán, Sinaloa Mexico; 4 Posgrado en Ciencias del Mar y Limnología, Universidad Nacional Autónoma de México, Av. Ciudad Universitaria 3000, 04510 Coyoacán, Ciudad de Mexico Mexico; 5 Facultad de Ciencias del Mar, Universidad Autónoma de Sinaloa, Paseo Claussen S/N Col. Centro, 82000 Mazatlán, Sinaloa Mexico; 6 Universidades del Bienestar Benito Juárez García, Buaysiacobe, Etchojoa, Sonora Mexico

**Keywords:** *Oreochromis aureus*, *Cyprinus carpio*, *Micropterus salmoides*, Heavy metals, Bioaccumulation, Biomagnification

## Abstract

During January 2013, a mining spill occurred in the Santa Maria mining region, releasing around 300,000 m^3^ of tailings on Los Remedios river, which was transported through the San Lorenzo river and finally to El Comedero (EC) dam. Twenty months later, we examined the concentrations of Hg and Se in the muscle, liver, gills, and guts of three fish species (*Cyprinus carpio*, *Oreochromis aureus*, *Micropterus salmoides*) captured in the EC dam to assess the performance of the cleaning operations. A high Se concentration in the liver of all species (carp, 1.2 ± 0.4; tilapia, 3.9 ± 2.1; bass, 3.5 ± 1.1 µg g^−1^ ww) was consistently observed, while this behavior was only found in the blue tilapia for Hg (0.15 ± 0.11 µg g^−1^ ww). Tilapia (benthic-detritivorous) exhibited the highest Se concentrations compared to the carp (omnivore) and the largemouth bass (piscivore). In contrast, the largemouth bass had the highest Hg levels in the muscle compared with the other fishes. Such differences could be related to the different metabolism and feeding habits among species. Compared to a tilapia study carried out three months after the mine spill during a mortality event, a decrease was evident in the liver for Se and Hg by 7.2 and 4.7 times, respectively. This reveals that cleaning operations were more efficient for Se and less for Hg, and that a prolonged period was required for the partial recovery of the element levels in fish from sites impacted by mining. Considering the Mexican consumption scenarios for each fish species, it could be concluded that there will be no non-cancer risk by exposure to Hg or Se.

## Introduction

Mercury is one of the most common contaminants that induce poisoning in biota and humans; its bioaccumulation leads to a diversity of toxic effects on various organs and tissues. It enters the aquatic environment through both natural and anthropogenic sources, where it is frequently biomagnified in the food chain (Páez-Osuna et al. 2017). In general, fish are more vulnerable to the severe toxicity of Hg as they are at an intermediate or higher trophic level in the aquatic food web (Molina-García et al. 2021); Hg interferes with the expression of proteins and enzymes; compromises important pathways, such as apoptosis and glucose metabolism; and induces the expression of metallothioneins (Souza Vieira et al. 2023). The consumption of fish that contain Hg becomes a relevant source of exposure in humans. Consequently, fish have been widely recognized as a significant dietary source of Hg exposure for coastal populations and high consumers during the last four decades, receiving continuous attention from health institutions and scientists (Ruelas-Inzunza et al. 2020). Conversely, Se plays a vital role in biogeochemistry and is an essential element in organisms; it is required for normal growth and development due to a cofactor of enzymes (e.g*.*, glutathione peroxidase or thioredoxin reductase) (Molina-García et al. 2021). Despite such beneficial features, elevated Se concentrations can biomagnify throughout food webs and result in toxic effects (Páez-Osuna et al. 2017). A well-known impact is that Se induces cytotoxicity and genotoxicity through the generation of reactive oxygen species (ROS) (Ali et al. 2021). An essential factor in evaluating the risk associated with Hg exposure is its interaction with Se, that Se exerts a protective effect against Hg toxicity. Numerous studies indicate that Se and Hg behave antagonistically, so their co-occurrence reduces their toxic effects. However, the outcome strongly depends on the chemical forms and molar ratio of these elements (Ralston et al. 2007; Branco et al. 2012; Molina-García et al. 2021).

The recent accelerated development of the economy leads to the high demand for metal(loid)s and their compounds, which are indispensable components in a wide range of everyday products such as construction materials, vehicles, computers, telephones, and paints (Kossoff et al. 2014). Consequently, mining has been rapidly developing during the last century resulting in considerable emissions and discharge of metal(loid)s. Mining produces massive volumes of waste, mainly tailings, which are often stored in impoundment dams; however, these dams can fail and have subsequent environmental, economic, and human health impacts (Kossoff et al. 2014). The chemical composition of tailings depends on the mineralogy of the ore body, the processing fluids nature, the extraction process’s efficiency, and the degree of weathering during storage in the impoundment. Various metal(loid)s are present in tailings since no extraction process reaches 100% efficiency, in which As, Cu, Cd, Hg, Se, and Zn are generally present in elevated concentrations (Páez-Osuna et al. 2022).

**Fig. 1:**
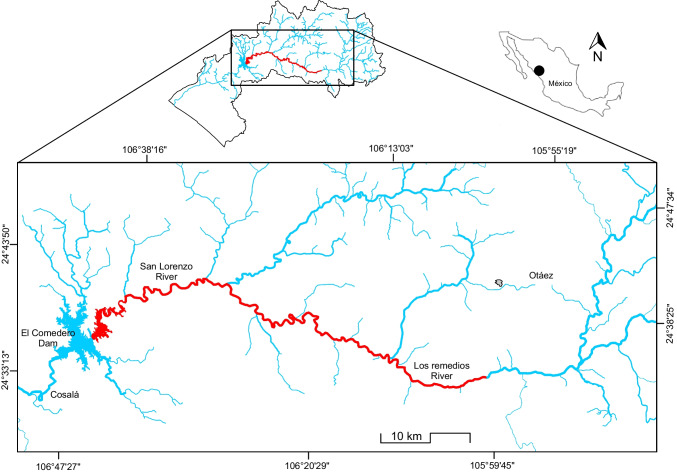
Illustration of the spill-affected zone along Los Remedios River-San Lorenzo River (red); the right extreme corresponds to the discharge site where the mine-tailings dam failure occurred

In the northwest Mexico, mining is a traditional economic activity mainly dedicated to producing Ag and Au. In the surroundings of the Gulf of California, particularly in the Baja California Sur, Sonora, and Sinaloa, numerous sites of mining interest were or are being exploited (Páez-Osuna et al. 2017, 2022). Unexpectedly, nine accidents occurred during 2013–2021 (dam failures and leaks) with a variable magnitude between 300 and 300,000 m^3^, most of which originated on the gulf’s continental margin (Páez-Osuna et al. 2017, 2022). In the particular case of the San Lorenzo basin located in Sinaloa and Durango, a mine spill (~ 300,000 m^3^) affected Los Remedios (LR) River (main tributary of San Lorenzo River), upper San Lorenzo River, and El Comedero (EC) dam (Fig. 1) in January 2013. This significantly impacted the waters and suspended sediments (Páez-Osuna et al. 2015), causing massive fish mortality (Páez-Osuna et al. 2022). In the subsequent week of the spill, the condition changed in the affected section (Fig. 1); an emergency soil clean-up procedure was developed after the accident, and the sludge covering the discharge site of LR River was mechanically removed from most of the affected land. Despite these cleaning operations, it is anticipatable that the affected area could show contamination levels by Hg and other elements associated with the mine spill.

In a previous study (Páez-Osuna et al. 2022), the concentration of six metal(loid)s in the fish *Oreochromis aureus* from EC dam was examined during a massive mortality event that occurred 3 months after the mine tailing spill. Higher levels of As, Cd, Cu, Hg, Se, and Zn were found in the liver, revealing that fish were exposed to high concentrations of these elements. In the present study, we examined a set of samples from three fish species of EC dam to assess the accumulation of Hg and Se in the muscle, gill, liver, and gut of the common carp *Cyprinus carpio*, the blue tilapia *O. aureus*, and the largemouth bass *Micropterus salmoides*, 20 months after the mine spill (~ 17 months after the massive fish mortality). We tested the hypothesis that three fish species with different feeding habits exhibit variable accumulation of Hg and Se in an ecosystem previously affected by a mine-tailing spill. A second hypothesis is that a reduction of Hg and Se concentrations in fish should be reached 20 months after mine spill (17 months after massive mortality); and, finally, a complimentary hypothesis is that the reduction of Hg and Se must be sufficient so that the muscle does not represent a risk to the health of consumers. Thus, the aims of this study are (i) to determine the concentration of Hg and Se in the three fish species to evaluate the differences among fishes and tissues; (ii) to assess the performance of cleaning operations and pollution status through the fish *O. aureus* growing in the spill-affected dam 20 months after the accident, as well as to compare these results with those obtained in *O. aureus* during the massive mortality that occurred 3 months after the mine spill; and (iii) finally, to evaluate the potential health risk for humans that consume the muscle of these three fish species.

## Materials and methods

### Study area and sampling

El-Comedero dam, located (24° 30′ N; 106° 45′ W) in the southeastern Gulf of California (NW Mexico), has a surface ~ 9200 ha and a volume between 400 and 1900 Mm^3^, where depth can reach 70 m (Fig. 1). EC dam receives waters from the upper San Lorenzo River, which is formed in the Sierra Madre Occidental; one of its main tributaries is LR River, which received the discharge directly from the mining spill. Three fish species, including the common carp *C. carpio*, the largemouth bass *M. Salmoides*, and the blue tilapia *O. aureus*, were introduced into EC dam for economic, alimentary, and touristic purposes (Beltrán Álvarez et al. 2015). A total of 45 fish were collected in EC dam, including *M. salmoides* (*n* = 22), *O. aureus* (*n* = 16), and *C. carpio* (*n* = 7) (Table 1). Each specimen was measured, weighed, and dissected to separate the liver, gills, guts, and a portion of the muscle. The separated fish tissues were well kept in a freezer for posterior laboratory analysis.

**Table 1:** Morphometric variables of fish species caught in El Comedero dam

Species	Total length (cm)			Weight (g)		
	Min	Max	Mean ± SD	Min	Max	Mean ± SD
*C. carpio* (n = 7)	36.5	47.0	40.3 ± 4.1 c	625	1725	1002 ± 400 c
*O. aureus* (n = 22)	21.0	34.0	26.3 ± 2.7 a	165	670	315 ± 102 a
*M. salmoides* (n = 16)	24.0	38.0	31.9 ± 4.1 b	170	740	490 ± 181 b

Different letters indicate significantly different (*p* < 0.05) mean concentrations between the variables of the fish species

*SD* standard deviation, *n* number of individuals

### Chemical analysis

All tissues were lyophilized (72 h, − 55 °C and 75 × 10^−3^ mbar), pulverized, and homogenized in a semiautomatic agate mortar. The digestion (5 mL of concentrated (~ 70%) nitric acid, Instra-analyzed J.T. Baker) of duplicate aliquots (~ 300 mg) was carried out using Teflon vials (Savillex) at 125 °C for 3 h (Bergés-Tiznado et al. 2015; Páez-Osuna et al. 2022). The livers were digested using 2 mL of H_2_O_2_ (30%) and 3 mL of concentrated nitric acid. The analysis of Se was carried out by atomic absorption spectrophotometry (AAS) with Zeeman correction background effect coupled to a graphite furnace oven (AAnalyst 800, PerkinElmer). A matrix modifier, a solution of Pd(HNO_3_)_2_ and Mg(NO_3_)_2_, was used in each sample atomization for this metalloid. Mercury was determined by AAS coupled to a cold vapor generator. Before Hg analysis, the samples were prepared by adding HNO_3_ (50%) and K_2_Cr_2_O_7_ (1%). The accuracy of the employed procedure was assessed with a certified reference material DOLT-4 (dogfish liver, NRC-CNRC 2008). Recoveries were 93.3 ± 6.3% for Hg and 106.5 ± 3.8% for Se, and precision fluctuated from 3.6 to 5.5% for Se to 6.8 to 8.1% for Hg. One blank was analyzed for every ten samples using the same procedure to test for contamination.

### Risk assessment

The Se/Hg molar ratio was calculated from individual results of Se and Hg of each tissue divided by the molecular weight of each element. The Se health benefit value (HBV_Se_) was calculated for edible muscle with the equation (Ralston et al. 2016): HBV_Se_ = ([Se − Hg] / Se) × (Se + Hg). Positive results indicate that Se exceeds Hg and benefits consumers; negative values mean the contrary (Ruelas-Inzunza et al. 2020). The magnitude of the value indicates Se surplus or deficit related to the theoretical consumption of fish muscle.

The non-cancer risk assessments were calculated by comparing an estimate of exposure to a reference dose (RfD) for oral exposures (EPA 2005) using the individual target hazard quotient (THQ) and the sum of THQs as the hazard index (HI): THQ = [EF × ED × FIR × C / RfD × BW × AT] × 10^−3^ and HI = ΣTHQ (Páez-Osuna et al. 2022). EF is an exposure frequency of 365 days year^−1^; ED is a 70-year exposure period; C is the mean concentration of the element (mg kg^−1^); BW is the population body weight of 75, 65, and 20 kg for adult men, female, and children (3–5 years old), respectively; and AT is the average exposure of 25,500 days. FIR is the food ingestion rate under different scenarios based on the consumption of fish and shellfish per capita in Mexico during 2021 (SEMARNAT 2022). A specific tilapia consumption of 11.5 g week^−1^ (1.6 g day^−1^) was utilized, followed by carp consumption of 1.2 g week^−1^ (0.2 g day^−1^), and largemouth bass consumption of 16.1 g week^−1^ (2.3 g day^−1^), corresponding to the amount consumed for other non-official registered species. Finally, a global consumption of 10.83 kg per capita (ration of 207.7 g week^−1^) was also used to assess the non-cancer risks. There would be a risk if THQ or HI > 1; additionally, the RfD data for Hg (0.0001 mg kg BW^−1^ day^−1^) and Se (0.005 mg kg BW^−1^ day^−1^) were obtained from the IRIS Assessment Base (EPA 2023). It is important to notice that the total Hg average as methyl-Hg was assumed to be conservative about risks. Finally, a safe intake or food ingestion rate (FIR) was calculated assuming THQ = 1 for the three species and two elements.

### Data analysis

The databases were completed in Excel, and the variables were tested using STATISTICA (version 7, StatSoft Inc.). The data were normally distributed and homoscedastic. The results were statistically compared between tissues, species, elements, and molar ratios by a one-way ANOVA and Tukey post hoc tests. The associations or correlations established among the variables were assessed by a product-moment correlations test yielding an *r* statistic.

## Results and discussion

The present study was carried out 20 months after the mine spill (~ 17 months after massive fish mortality). The tailing spill occurred ~ 150 km from EC dam on January 21, 2013, and the massive fish mortality emerged ~ 90 days later. Considering the morphology and current velocities, it is probable that the spilled material was transported in ~ 35 days from the site of the spill to EC dam (Páez-Osuna et al. 2022). During the subsequent days of the spill, an emergency tailing cleaning procedure was applied, and the tailing-sludge was mechanically removed from most of the affected areas in LR River. However, the affected zone could still present pollution by metal(loid)s even after this cleaning procedure.

### Mercury and selenium in fish tissues

Specimens of the sampled fish species exhibited variable sizes, corresponding to pre-adults and adults (Table 1). However, the total length (*F* = 46.3, *p* < 0.05) and the weight (*F* = 31.5, *p* < 0.05) were different among species. In general, element concentrations exhibited moderate variability in the tissues. Selenium was consistently higher in the liver and lower in the guts and muscle of the three fish species (Table 2). Conversely, Hg was highest in the liver of the blue tilapia, while Hg was more elevated in the muscle of the common carp and largemouth bass; the lowest levels were evidenced in the guts of these two fish species. However, the differences in Hg concentrations among the tissues of the three species were relatively small, although the exception could be the relatively high concentrations in the muscle of the largemouth bass (Table 2).

**Table 2:** Total mercury and selenium (mean ± SD, wet weight) concentrations and molar ratios in tissues species caught in El Comedero dam

Tissue	Se	Se	Hg	Hg	HBV_Se_	Molar ratio	
	µg g^−1^	nmol g^−1^	µg g^−1^	nmol g^−1^		Se/Hg	Hg/Se
Common carp							
Muscle	0.7 ± 0.3a	8.6 ± 3.6	0.09 ± 0.06b	0.4 ± 0.3	8.5 ± 3.7a	26.0 ± 14.7a	0.1 ± 0.1
Liver	1.2 ± 0.4b	14.6 ± 5.2	0.04 ± 0.03a,b	0.2 ± 0.2	14.6 ± 5.2b	92.4 ± 35.2a,b	< 0.1
Gills	0.8 ± 0.3a,b	9.6 ± 3.4	0.02 ± 0.01a	0.1 ± 0.1	9.6 ± 3.4a,b	118.3 ± 67.5b	< 0.1
Guts	0.6 ± 0.2a	7.3 ± 2.3	0.02 ± 0.01a	0.1 ± 0.1	7.3 ± 2.3a	107.7 ± 88.3a,b	< 0.1
Blue tilapia							
Muscle	0.6 ± 0.2a,b	7.7 ± 2.3	0.07 ± 0.05a	0.4 ± 0.2	7.7 ± 2.3a,b	29.2 ± 17.3a	< 0.1
Liver	3.9 ± 2.1c	49.7 ± 26.0	0.15 ± 0.11b	0.8 ± 0.6	49.7 ± 26.0c	93.7 ± 74.5b	< 0.1
Gills	1.4 ± 0.4b	18.1 ± 5.4	0.03 ± 0.01a	0.1 ± 0.1	18.1 ± 5.4b	135.3 ± 47.6c	< 0.1
Guts	0.5 ± 0.2a	6.4 ± 2.8	0.07 ± 0.03a	0.3 ± 0.1	6.4 ± 2.8a	20.8 ± 11.5a	0.1 ± 0.0
Largemouth bass							
Muscle	0.8 ± 0.3a	10.7 ± 3.9	0.38 ± 0.16b	1.9 ± 0.8	10.2 ± 4.2a	10.4 ± 12.9a	0.2 ± 0.1
Liver	3.5 ± 1.1c	44.0 ± 13.6	0.15 ± 0.06a	0.8 ± 0.3	43.9 ± 13.6c	70.6 ± 40.3c	< 0.1
Gills	1.8 ± 0.6b	22.6 ± 7.1	0.13 ± 0.07a	0.6 ± 0.4	22.6 ± 7.1b	50.7 ± 34.8b,c	< 0.1
Guts	0.9 ± 0.3a	11.8 ± 3.8	0.11 ± 0.06a	0.5 ± 0.3	11.8 ± 3.7a	31.1 ± 20.6a,b	< 0.1

Different letter indicates significantly different (*p* < 0.05) mean concentrations between tissues of each species element

*SD* standard deviation

Common carp. Hg had significantly higher accumulation in the muscle (*F* = 5.5, *p* < 0.05), ranging from 0.04 to 0.23 µg g^−1^ (ww), followed by the liver (0.01–0.11 µg g^−1^ ww), gills, and guts (0.01–0.05 µg g^−1^ ww) (Table 2). The liver of the carp had the significantly highest (0.76–2.00 µg g^−1^ ww) concentrations of Se (*F* = 5.2, *p* < 0.05) in comparison to the other tissues, followed by the gills (0.39–1.20 µg g^−1^ ww), muscle (0.31–1.08 µg g^−1^ ww), and guts (0.40–0.97 µg g^−1^ ww) (Table 2). Regarding the Se/Hg, values were > 1.0 in the four tissues, and the highest was found in the gills and the lowest in muscle, significantly different among muscle and the other three tissues (*F* = 3.5, *p* < 0.05). The HBV_Se_ varied significantly (*F* = 5.2, *p* < 0.05) in descending order liver > gills > muscle > guts (Table 2).

#### Blue tilapia

Hg showed higher accumulation in the liver (range 0.01–0.48 µg g^−1^ ww) (*F* = 14.8, *p* < 0.05), followed by the muscle (0.02–0.22 µg g^−1^ ww), guts (0.02–0.15 µg g^−1^ ww), and gills (0.01–0.07 µg g^−1^ ww), with comparable means (*p* > 0.05) (Table 2). Regarding Se levels, the highest mean concentrations were found in the liver (*F* = 50.0, *p* < 0.05) ranging from 1.55 to 10.05 µg g^−1^, followed by the gills (0.67–2.34 µg g^−1^ ww), muscle (0.37–1.14 µg g^−1^ ww), and the lowest in the guts (0.24–1.05 µg g^−1^ ww). The molar Se/Hg ratios showed differences (*F* = 32.2, *p* < 0.05) between the tissues means of tilapia; the highest were found in the gills (66.1–232.2), followed by the liver (22.2–347.9), muscle (7.9–80.1), and guts (7.6–48.7 ww); means of Se/Hg > 1 for all the tissues. The latter was also observed for the HBV_Se_, with statistically higher (*F* = 50.0, *p* < 0.05) positive values in the liver than in the gills and guts, following the same behavior as the common carp.

#### Largemouth bass

The mean concentrations of Hg among the largemouth bass tissues were significantly different (*F* = 25.9, *p* < 0.05) between the muscle and other tissues (Table 2), with higher levels in the muscle (0.07–0.56 µg g^−1^ ww), followed by the liver (0.03–0.24 µg g^−1^ ww), gills (0.03–0.33 µg g^−1^ ww), and guts (0.02–0.24 µg g^−1^ ww). As with the other two species, the levels of Se in the liver were the highest (1.43–5.38 µg g^−1^ ww) and significantly different (*F* = 57.6, *p* < 0.05) from the gills (1.17–2.99 µg g^−1^ ww), guts (0.65–1.76 µg g^−1^ ww), and muscle (0.46–1.38 µg g^−1^ ww). The average Se/Hg molar ratios in the tissues of the largemouth bass were different (*F* = 12.5, *p* < 0.05), and the highest were found in the liver (32.4–201.2), followed by the gills (10.2–114.4), guts (12.1–79.0), and muscle (2.8–45.3) (Table 2). The HBV_Se_ values were higher (*F* = 58.0, *p* < 0.05) positive values in the liver > gills > guts > muscle (Table 2).

The accumulation pattern in the tissues was different in the three fish species; a high concentration was consistently observed in the liver of all fish species, while this behavior was only found for Hg in the tilapia. The pattern in which the liver accumulates higher metal(loid) levels has been typically observed in numerous studies regarding freshwater (Yap et al. 2015; Páez-Osuna et al. 2022) and marine (Ruelas-Inzunza et al. 2011, 2020) species. In addition to the organ specificity in the uptake, storage, regulation, and excretion abilities, the different types of exposure associated with the feeding habits of each fish species are also important. Tilapia is a predominantly omnivore benthic species that consume phytoplankton, zooplankton, copepods, cladocerans, small invertebrates, and detritus (Froese and Pauly 2022). This species exhibited the highest Se concentrations in the liver compared to the carp, an omnivore that mainly consumes plankton, fish larvae, and plants (Froese and Pauly 2022), but similar to Se in the liver of the largemouth bass. Nevertheless, the largemouth bass had higher Se accumulation in the other tissues. The latter might be related to this species diet, which includes fishes and crustaceans, and it can also be cannibalistic (Froese and Pauly 2022).

In contrast, the largemouth bass had the highest Hg levels in the muscle, gills, and guts compared with other fish species. Interestingly, the liver of the tilapia and the largemouth bass accumulated the same Hg mean concentration (Table 2). The high accumulation in the liver is related to the capture and assimilation of metal(loid)s through food and water, as it is directly associated with metabolism and respiration (Ruelas-Inzunza et al. 2011). The ability of the liver to accumulate these elements is a result of the activity of the metallothioneins, which interact with these elements reducing their toxicity (Páez-Osuna et al. 2022). The metallothionein induction in fish is high in organ tissues such as the liver and kidney, which are involved in metal(loid) uptake, storage, and excretion (Viarengo et al. 2007).

The tilapia results suggest that the liver of *O. aureus* is highly active in the uptake, storage, and detoxification of Se and perhaps moderately active for Hg. Therefore, this organ has been considered useful as a potential biomonitor of metal pollution since liver concentrations could be proportional to those in the environment (Yap et al. 2015). However, it is important to mention that the blue tilapia could be particularly useful for monitoring metal(loid) bioavailability in the detritus and the benthic environment where this fish generally feeds.

A decrease was observed in the values found in the liver of *O. aureus* for both metal(loid)s: Se 152 ± 46 and Hg 3.81 ± 1.21 µg g^−1^ dw in April 2013, 3 months after the mine spill (Páez-Osuna et al. 2022) versus the levels found in the present study, 20 months after the mine spill: Se 21.1 ± 11.3, and Hg 0.81 ± 0.59 µg g^−1^ dw. Therefore, Se decreased 7.2 ± 4.0 times and Hg 4.7 ± 2.0 times. This indicates that the cleaning operations and the natural depuration performance were more efficient for Se but less for Hg. The baseline levels in *O. aureus* are unavailable in the study area; however, an experimental study indicates that the control liver accumulates 0.31 µg Hg g^−1^ (Allen 1994). Therefore, the Hg found (Hg 0.81 ± 0.59 µg g^−1^) 20 months after the mine-tailing spill is still relatively high.

In general, the concentrations of Se and Hg were higher in the piscivore fish (largemouth bass) than in the other fishes. This could be related to the different feeding habits of the three fish species; the carp is omnivorous but feeds on a variety of benthic organisms and plants (Froese and Pauly 2022), exhibiting the lowest Hg level in the guts. Given that the largemouth bass is at the top of the food chain of EC dam, it possibly reflects the biomagnification of Se and Hg in the guts (diets). However, once Se and Hg are ingested, the uptake occurs in the intestines through membranes via transporter proteins or/and ionic channels in the studied fishes (Le Croizier et al. 2018). Thus, dietary accumulation occurs first in the digestive tract; after reaching the liver, both metal(loid)s are released into the general blood circulation system and finally reach secondary accumulation organs, such as the muscle. However, Hg accumulates higher in the liver of a piscivore fish, contrary to the blue tilapia, in which these two metal(loid)s are primarily accumulated in the liver compared to the muscle. This contrasting behavior reveals the different metabolism involved in three fish species with distinctive feeding habits.

### Metal(loid)s and biological parameter correlations

**Fig. 2:**
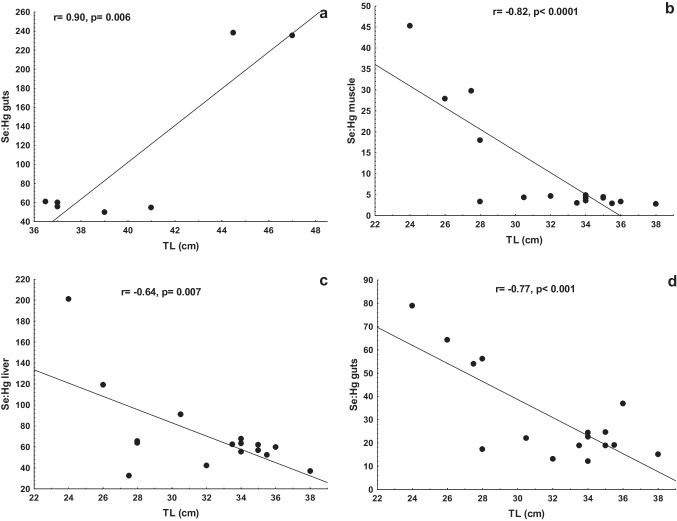
Variation of Se/Hg molar ratios with length (TL) in guts of the carp (**a**), and muscle (**b**), liver (**c**), and guts (**d**) of the largemouth bass

**Fig. 3:**
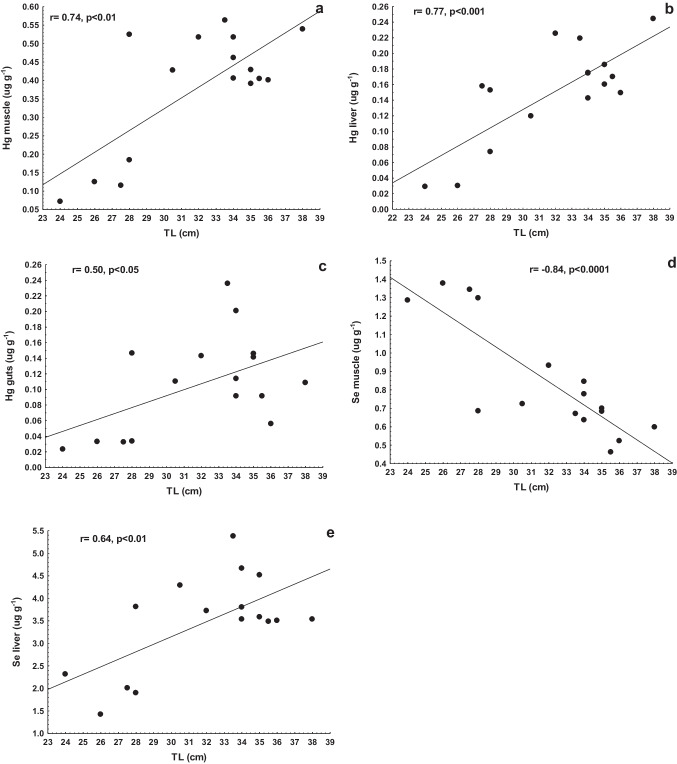
Variation of the concentration of Hg in muscle (**a**), liver (**b**), and guts (**c**) with length (TL), as well as Se in muscle (**d**) and liver (**e**) with the size of the largemouth bass

**Fig. 4:**
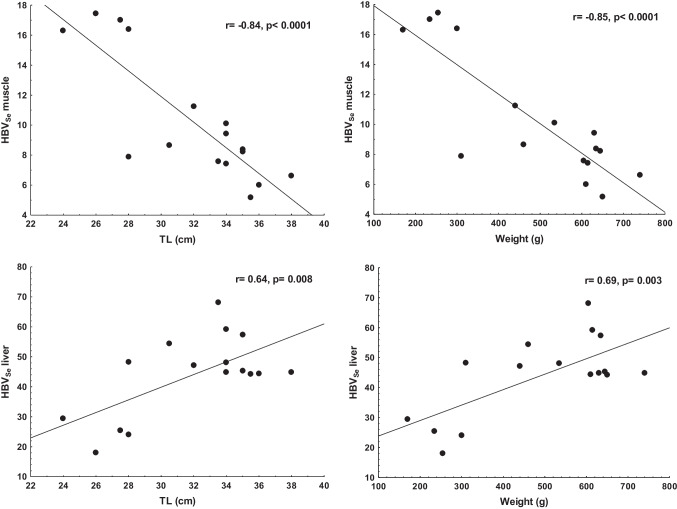
Variation of HBV_Se_ in– the muscle and liver with length and weight of the largemouth bass

The morphometric variables of TL and weight in the common carp were not significantly correlated (*p* > 0.05) to the measured elements in the studied tissues. The Se/Hg molar ratio data in the guts (*r* = 0.90) and gills (*r* = 0.77) were positively correlated (*p* < 0.05) to the common carp`s weight; TL was correlated (*r* = 0.90, *p* < 0.01) to Se/Hg molar ratio in the carp’s guts (Fig. 2a). The TL and weight were not statistically correlated to the measured Hg and Se in the tissues of the blue tilapia. None of the blue tilapia biometric data was significantly associated (*p* > 0.05) with the measured elements in the studied tissues. TL was correlated to Hg in the muscle, liver, and guts of the largemouth bass (Fig. 3a–c) and Se in the muscle and liver (Fig. 3d, e). The same significant correlations (*p* < 0.05) were found between the weight and Hg in muscle (*r* = 0.73), liver (*r* = 0.71), and guts (*r* = 0.53), as well with Se in the muscle (*r* =  − 0.86) and liver (*r* = 0.69) of the largemouth bass. The Se/Hg molar ratios in the muscle, liver, and guts were negatively correlated (*p* < 0.05) to the TL and weight of the organisms (Fig. 2b–d). Interestingly, when the largemouth bass measures between 28 to 38 cm (310 to 760 g), the Se/Hg in the muscle remains relatively constant. Similarly, there was a significant correlation (*p* < 0.05) between HBV_Se_ and the body size and weight of the largemouth bass (Fig. 4), negative for muscle and positive for the liver. This indicates a transference of Se to Hg from the muscle to the liver as individuals grow and gain weight (age).

The size effect on the accumulation of Hg and Se may be a function of any age-dependent parameters, such as changes in metabolism with age, different stages, or feeding habit differences (Páez-Osuna et al. 2022). Concentrations of Hg typically increase with fish age when the rate of dietary uptake is faster than elimination (Chételat et al. 2020). In the largemouth bass (piscivore species), the TL was positively correlated to Hg in the muscle, liver, and guts, and Se in the liver (Fig. 3a, b, c, e). Young mature individuals tend to consume more and larger fish prey, leading to bioaccumulation of Hg and Se which results in biomagnification. Conversely, the levels of Se in the muscle were negatively correlated to TL, showing a decreasing pattern as the individuals become larger, which can be related to the feeding habit differences between older and younger individuals (Páez-Osuna et al. 1995). This behavior has been observed in other species, such as sharks; growing processes often allow a higher Hg intake from larger prey, and Hg levels also typically increase proportionally with the predator’s body size (Lyons et al. 2013).

### Comparison with other regions

Ideally, metal(loid) concentrations should be compared with organisms of the same stage, age, size, and sex. However, it is difficult since, in most studies, the sampled and available organisms exhibit heterogeneous characteristics, and frequently the studies do not determine either age/stage, or sex. In the present discussion, we compiled metal(loid) data for the same species of the common carp and bass. In contrast, data from the same genera as the tilapia whose feeding habit is similar were included. In addition, succinct information was also included on the type of pollution present in the region where the fish were collected to enhance the discussion. The comparison table data is shown in dry weight (dw) to present a homogenized summary.

Concentrations of Hg and Se in the tissues of the common carp found in this study were contrasted with those reported in other areas (Table 3). Compared to Hg, data on Se are limited for this fish, although it is clear that there needs to be more consistency regarding levels in the muscle and liver. It is evident that the carp of EC dam exhibit intermediate concentrations in both tissues; the carp from the Keban dam (Turkey) and those from Tai and Baiyangdian lakes (China) showed higher concentrations of Se, where a chrome factory (Danabas et al. 2020), as well as industrial and agricultural effluents, is present (Zhang et al. 2019a). The case of Hg in the muscle and liver was similar; the carp from EC dam exhibited low or intermediate levels compared with most compiled studies (Table 3).

**Table 3:** Ranges and mean concentration (µg g^−1^ dw) of mercury and selenium in the common carp around the world

Tissue	Se	Hg	Type of pollution	Region	Reference
Muscle Liver	- -	0.23 ± 0.03 0.92 ± 0.14	Mining and heavy industrial	Jinsha River, Yangtze River, China	Li et al. (2018)
Muscle Liver	- -	0.23 ± 0.04 0.13 ± 0.01	Small number of industries	Tuojiang River, Yangtze, River, China	Li et al. (2018)
Muscle Liver	4.6–8.4 4.3–6.5	7.7–8.4 7.9–8.6	Chrome factory	Keban dam, Turkey	Danabas et al. (2020)
Muscle	-	0.10–0.23	Eutrophic	Wujiangdu dam, SW China	Jing et al. (2021)
Muscle	-	2.74 ± 1.49	Limited contamination	Wetlands of Yellow River Delta, China	Cui et al. (2011)
Muscle	-	0.54 ± 0.30	Mining, domestic wastewater, agriculture	Upper Mekong River, China	Zhang et al. (2019a)
Muscle	6.8	0.037	Atmospheric deposition, industrial and agricultural effluents	Tai and Baiyangdian Lakes, China	Zhang et al. (2019b)
Muscle	0.069–5.89	0.031–0.366	Agriculture, wastewater treatment plants	Tablas de Daimiel Park, Spain	Fernández-Trujillo et al. (2021)
Muscle Liver	- -	2.86–4.76 1.67–2.98	Agriculture	Busko Blato Reservoir, Bosnia and Herzegovina	Has-Schon et al. (2015)
Muscle	-	0.095 ± 0.018	Atmospheric deposition, agricultural, and urban emissions	Trebon region, Czech Republic	Kral et al. (2017)
Muscle	-	0.083–0.143	Limited pollution (towns and villages)	Shadegan I. wetland, Iran	Rahmanikhah et al. (2020)
Muscle Liver	- -	0.14 ± 0.02 0.58 ± 0.09	Mining, heavy industry	Jinsha River, China	Li et al. (2018)
Muscle Liver	- -	0.14 ± 0.02 0.50 ± 0.05	Small number of industries	Tuo River, China	Li et al. (2018)
Muscle	-	0.46 ± 0.33	Wastewater streams	Lake Zapotlán, Mexico	Malczyk and Branfireun (2015)
Muscle Liver	3.3 ± 1.4 4.2 ± 1.4	0.42 ± 0.28 0.14 ± 0.10	Mining tailing spill (after 20 months)	El Comedero dam, NW Mexico	This study

-, not analyzed; moisture levels considered to change from wet weight to dry weight, muscle 83.2%, liver 80.5%, and guts 74.3%

Regarding the blue tilapia, the information on Se is also limited. However, the levels found in the tilapia from EC dam are high compared to those registered in most studies (Table 4). Only the high levels of Se in the liver (152 ± 46 µg g^−1^ dw) previously reported in EC dam during the mass mortality event (Páez-Osuna et al. 2022) are remarkable, as they stand out from any other concentration reported (Table 4). The Hg levels in the muscle and liver of the blue tilapia showed intermediate concentrations. These concentrations are low compared to the tilapias (*O. niloticus* and *Tilapia zillii*) from contaminated sites such as the Barekese dam (Ghana) and the wastewater ponds (Egypt), which are influenced by artisanal mining (Gymah et al. 2018) and wastewater (Khallaf et al. 2003), respectively (Table 4).

**Table 4:** Ranges and mean concentration (µg g^−1^ dw) of selenium and mercury in tilapia around the world

Species	Se	Hg	Type of pollution	Region	Reference
*O. niloticus*					
Liver	-	47.8	Wastewater ponds	Shanawan canal, Al-Minufiya, Egypt	Khallaf et al. (2003)
*O. niloticus*					
Muscle	-	0.11–0.43	Agricultural and industrial	Manzala lake, Egypt	Sallam et al. (2019)
*O. niloticus*					
Liver	9.8 (4.7–15.0)	-	Urban sewage and agriculture	Lake Phewam, Nepal	Rosseland et al. (2017)
*O. niloticus*					
Muscle	-	3.33	Artisanal mining and agriculture	Barekese dam, Ghana	Gymah et al. (2018)
*Tilapia zillii*					
Muscle	-	5.42			
*O. niloticus*					
Muscle	-	0.02–0.53	Domestic and industrial	Senegal River, Mauritania	El Mahmoud-Hamed et al. (2019)
*O. niloticus*					
Muscle	0.007–0.008	0.059–0.071	Industrial	Koka lake, Ethiopia	Dsikowitzky et al. (2013)
Liver	0.001–0.017	0.024–0.111			
*O. niloticus*					
Muscle	0.001–0.002	0.045–0.241	Textile, ceramics municipal	Awasa lake, Ethiopia	Dsikowitzky et al. (2013)
Liver	0.002–0.003	0.089–0.164			
*Sarotherodon melanotheron*					
Muscle	-	1.54	Agriculture, industrial	Awba dam, Nigeria	Adeogun et al. (2020)
*O. mossambicus*					
Muscle	-	< 0.1	Mining activities	Yonki dam, Papua New Guinea	Kapia et al. (2016)
*O. mossambicus*					Huang et al. (2003)
Muscle	-	-	As in groundwater	Farms SW coastal area Taiwan	
*O. mossambicus*					
Muscle	2.50 ± 0.36	-	As in groundwater	Farms south Taiwan	Lin et al. (2005)
*O. mossambicus*					
Muscle	23.5 ± 4.6	-	As in groundwater, industrial and agriculture	Farms west coast Taiwan	Ling et al. (2013)
*Oreochromis* spp*.* Muscle	-	0.21 ± 0.12	Wastewater streams	Lake Zapotlán, Mexico	Malczyk and Branfireun (2015)
*O. aureus*					
Muscle	-	0.12–0.36	Mining area	Picachos dam, NW Mexico	Ruelas-Inzunza et al. (2015)
Liver	-	0.57			
*O. aureus*					
Muscle	10.7 ± 0.4	0.32 ± 0.01	Mining tailing spill (mortality event)	El Comedero dam, NW Mexico	Páez-Osuna et al. (2022)
Liver	152 ± 46	3.81 ± 1.21			
*O. aureus*					
Muscle	3.2 ± 1.1	0.38 ± 0.27	Mining tailing spill (after 20 months)	El Comedero dam NW Mexico	This study
Liver	21.1 ± 11.3	0.81 ± 0.59			

-, not analyzed; moisture levels considered to change from wet weight to dry weight, muscle 83.2%, liver 80.5%, and guts 74.3%

Concerning the largemouth bass, the data on Se is also limited, particularly in the liver; in muscle, the Se concentrations from EC dam were intermediate compared to those from diverse regions with several types of contamination (Table 5). At the same time, Se in the liver was higher in the fishes of EC dam compared to those from Reed River (South Carolina, USA), which are influenced by agriculture and urbanism (Otter et al. 2012). The fish from EC dam exhibited intermediate concentrations of Hg in the muscle; high Hg levels correspond to fish from the Henderson Lake (Louisiana, USA), NW Florida rivers (USA), Sipsey River (Alabama, USA), and the Sacramento-San Joaquin Delta Region (California, USA), where atmospheric deposition, agriculture, municipal incinerators, coal-fired power plants, industry, and Au and Hg mining activity are present (Table 5).

**Table 5:** Ranges and mean concentration (µg g^−1^ dw) of mercury and selenium in the largemouth bass fish around the world

Tissue	Se	Hg	Type of pollution	Region	Reference
Muscle	-	3.16	Au and Hg mining activity	Sacramento-San Joaquin Delta Region, California, USA	Davis et al. (2008)
Muscle	-	5.18	Atmospheric deposition and industrial	Sipsey River, Alabama, USA	Prarthana and Findlay (2017)
Muscle	-	1.13	Atmospheric deposition	Black Warrior River, Alabama, USA	Prarthana and Findlay (2017)
Muscle	-	1.90–6.07	Atmospheric deposition, municipal incinerators, coal-fired power plants	NW Florida rivers, USA	Karouna-Renier et al. (2011)
Muscle	1.01–1.67	1.13–1.73	Atmospheric deposition, agriculture, and industry	Atchafalaya River, Louisiana, USA	Reyes-Avila et al. (2019)
Muscle	0.54–1.07	2.92–3.69	Atmospheric deposition, agriculture, and industry	Henderson Lake, Louisiana, USA	Reyes-Avila et al. (2019)
Muscle	-	1.96	Nuclear weapons production, atmospheric deposition, industry	Savannah River, South Carolina and Georgia, USA	Burger et al. (2002)
Muscle	-	0.51–0.53	Atmospheric deposition, agriculture, industry	Lower Mississippi River, USA	Watanabe et al. (2003)
Muscle	3.15–15.9	0.54–1.49	Agriculture and mining (coal, Cu, U)	Colorado River and its tributaries, USA	Hick et al. (2007)
Muscle	2.44–6.82	0.060–0.096	Agriculture, wastewater treatment plants	Tablas de Daimiel Park, Spain	Fernández-Trujillo et al. (2021)
Muscle Liver	0.9–1.3 4.2–6.6	- -	Agriculture, forest, urban	Reedy River watershed, South Carolina, USA	Otter et al. (2012)
Muscle	-	0.33–0.96	Domestic and industrial discharges, agriculture	Mechraa-Hammadi dam, Morocco	Mahjoub et al. (2021)
Muscle Liver	3.4 ± 1.3 15.7 ± 4.9	1.60 ± 0.67 0.67 ± 0.27	Mining tailing spill (after 20 months)	El Comedero dam, NW Mexico	This study

-, not analyzed; moisture levels considered to change from wet weight to dry weight, muscle 83.2%, liver 80.5%, and guts 74.3%

From this robust contrast, it is possible to generalize that the three fish species collected in EC dam 20 months after showed intermediate levels of Se and Hg in both the liver and muscle. The concentrations recorded in EC dam for the blue tilapia deserve particular attention since, in a previous study (Páez-Osuna et al. 2022), there was massive mortality of fish whose concentrations in the liver were extremely high (Table 4). Compared with this study, the decrease was marked 7.2 ± 4.0 times for Se and 4.7 ± 2.0 times for Hg.

### Risk assessment

From the perspective of human health by consuming the edible fraction of fish, there are a variety of criteria to discern acceptable levels. The fish fillet (muscle) is commonly the focus since it is the primary support of the human diet. In Mexico, the local human population consumes tilapia fillet produced nationally, which in 2018 was 116,000 t (FAO 2021), with an average consumption per capita of 0.60 kg in 2021 (SEMARNAT 2022). Therefore, it is crucial to generate information on tilapia fisheries occur in areas influenced by mining, as in NW Mexico. Thus, by considering the Mexican consumption scenarios for each species (Table 6), it could be said that there will be no non-cancer risk by exposure to Hg or Se. Nonetheless, if an edible portion of 207.7 g per week of blue tilapia or common carp is consumed, only children (20 kg BW) could be at Hg risk (THQ and HI > 1). Nevertheless, the hazard risk was evidenced for all the population strata if the same portion of 207.7 g is eaten in a week (Table 6). A safe weekly intake of blue tilapia muscle would be less than 196.9, 640.0, and 738.4 g, and 56.6, 509.1, and 587.4 g of common carp muscle for children, women, and men, respectively. These weekly meals concerning the bass must be reduced to less than 36.7 g for children, 119.4 g for women, and 137.8 g for men to avoid risks from Hg exposure. It must be noted that consuming the flesh of any of the studied species would represent no risk at all from Se exposure; instead, it could be a nutritional benefit.

**Table 6:** Non-cancer risk assessment by population group from specific and total fish per capita rations for blue tilapia, common carp, and largemouth bass; children BW = 20 kg, women BW = 65 kg, and men BW = 75 kg

Element	THQ blue tilapia (11.5 g week^−1^)			THQ common carp (1.2 g week^−1^)			THQ largemouth bass (16.1 g week^−1^)		
	Children	Women	Men	Children	Women	Men	Children	Women	Men
Hg	0.058	0.018	0.016	0.007	0.002	0.002	0.438	0.135	0.117
Se	0.010	0.003	0.003	0.001	< 0.001	< 0.001	0.020	0.006	0.005
**HI**	**0.068**	**0.021**	**0.019**	**0.008**	**0.002**	**0.002**	**0.458**	**0.141**	**0.122**
Total fish consumption rate									
Element	THQ blue tilapia (207.7 g week^−1^)			THQ common carp (207.7 g week^−1^)			THQ largemouth bass (207.7 g week^−1^)		
Hg	1.055	0.325	0.281	1.326	0.408	0.354	5.652	1.739	1.507
Se	0.181	0.056	0.048	0.202	0.062	0.054	0.252	0.078	0.067
**HI**	**1.236**	**0.380**	**0.330**	**1.528**	**0.470**	**0.408**	**5.904**	**1.817**	**1.574**

*BW* body weight

The Hg levels in the muscle of the three fish species were (Table 2) far below the maximum permissible limit (MPL) (1.0 µg g^−1^ ww as methyl-Hg (MeHg); Mexican norm NOM-242-SSA1-2009, DOF 2011). Regarding Se, all individuals of the blue tilapia, 50% of the individuals of the carp and 33% of the largemouth bass, were above the threshold (0.3 µg g^−1^ ww) for fish and fish products established in Chile (FAO 1983). Moreover, 100% of the muscle samples of the three fish species were below the limit of New Zealand (2.0 µg g^−1^ ww) for any foodstuff (FAO 1983). This Se criterion is inconsistent and should be considered with caution.

It is important to highlight that in fish, most Hg is MeHg. In contrast with inorganic Hg (Hg[II]), MeHg can readily accumulate in aquatic organisms due to its high assimilation efficiency and low efflux rate from the body. It is also widely recognized as the predominant Hg form in fish tissue (Wang and Wang 2018). For example, in tilapia, diverse MeHg contributions to total Hg have been reported: 82% in *Oreochromis niloticus* (Sharma et al. 2013) and 53% in *T. zillii* (Rahmanikhah et al. 2020). However, experimental studies have determined that different diets can modulate the trophic Hg transfer in fish and that MeHg bioaccumulation is influenced by food quality and quantity (Wang and Wang 2018).

The Se/Hg molar ratio in the four tissues of the three fish species were > 1 (Table 2), indicating that Se is incorporated in selenoproteins (Páez-Osuna et al. 2022). Due to the high affinity between Hg and Se, the formation of a Hg-Se complex has been suggested as the mechanism responsible for the protective effect of Se (Ralston et al. 2016). Comparatively, it was observed that the blue tilapia exhibited the highest values among the muscle of the three species; that is, the Se protection is greater in this species and less in the bass (Table 2). The variation of Se/Hg molar ratio with size was observed in the largemouth, which was negatively correlated (*p* < 0.05) with the TL and weight in the muscle, liver, and guts (Fig. 2). The antagonistic effect of Se on Hg has been explained (Branco et al. 2012); a resultant excess Se induces an amplified production of selenoproteins, with the selenocysteine in this protein acting as a trap for CH_3_Hg preventing its access to different organs. Also, Se binding CH_3_Hg during co-exposure enhances its excretion. Considering the highest values of the Se/Hg molar ratio of the four tissues in the three fish species, it is evident that the blue tilapia appears more efficient in this context.

The HBV_Se_ in the four tissues of the three fish species were positive (Table 2). Some results are unexpected given that these fishes were exposed in lesser or greater quantities to the remnants of the mining material transported from the spill point, and hypothetically could be used for human consumption. However, this consumption needs to be considered due to the possible levels of other materials and elements that could be accumulated in the fish and were not analyzed in the present study.

## Conclusions

This study is the first to track Hg and Se levels in exposed fish 3 and 20 months after a mining spill. The accumulation patterns of Hg and Se in the tissues differed in the three fish species, which confirms the hypothesis that fish with different feeding habits exhibit variable Hg and Se accumulation. These results highlight that body size, habitat use, and feeding habits contribute to defining the different patterns of Hg accumulations in the three fish species. Regarding the largemouth bass, it is deduced that diet shifts towards higher Hg content prey items increased Hg accumulation rates in larger fish. Compared to a study conducted 90 days after the mine spill during a massive mortality of tilapia in EC dam (Páez-Osuna et al. 2022), Se and Hg decreased in the liver 7.2 ± 4.0 and 4.7 ± 2.0 times, respectively, 20 months after the spill (present study). This confirms the second hypothesis; a reduction of Se and Hg concentrations in fish should be reached after the mine spill. These results have important implications, because they indicate the prolonged time required for partial recovery of element levels in fish from a site impacted by mining.

The Se/Hg molar ratio in the four tissues of the three fish species were > 1, indicating Se’s protective role on Hg, which is more efficient in the blue tilapia. Conversely, the HBV_Se_ in the four tissues of the three fish species were positive, indicating that Se exceeds Hg and is beneficial to consumers. The safe weekly intakes proposed for children were less than 196.9, 156.6, and 36.7 g for the muscle of blue tilapia, common carp, and largemouth bass, respectively. The ration per week recommended for the blue tilapia would be 640.0 and 738.4 g, for common carp 509.1 and 587.4 g, and for largemouth bass 119.4 and 137.8 g, for women and men, respectively. These rations are recommended considering that other materials associated with the mining spill are absent or harmless. It is highly suggested that many biota species and samples be used for the best evaluation of the performance of the cleaning operations after the mine-tailing spill. The reduced number of fish species and samples is a weakness of this work, though the results are optimistic. Two research needs are identified from this study: the first is related to the examination of the changes in the biodiversity and other ecological impacts in the study area during different periods after the mine-tailing spill, and the second is to examine MeHg in these three fish species to more precisely quantify health risks for consumers.

## Data Availability

All data generated or analyzed during this study are included in this published article.
